# Ostéomyélite chronique compliquée d’un carcinome épidermoïde: à propos d’une observation

**DOI:** 10.11604/pamj.2017.28.188.13741

**Published:** 2017-10-31

**Authors:** Moez Hamdani, Ali Houidi, Amine Briki, Naoufel Haddad, Abdelmajid Khabir

**Affiliations:** 1 Laboratoire d’Anatomie et de Cytologie Pathologiques, Hôpital Habib Bourguiba - Médenine, Médenine, Tunisie; 2 Service d’Orthopédie, Hôpital Habib Bourguiba - Médenine, Médenine, Tunisie

**Keywords:** Ostéomyélite chronique, carcinome épidermoïde, fémur, transformation maligne, amputation, Chronic osteomyelitis, squamous cell carcinoma, femur, malignant transformation, amputation

## Abstract

La transformation maligne d’une ostéomyélite chronique est une complication rare et tardive qui survient le plus souvent au niveau des berges d’un trajet fistuleux avec extension et infiltration des tissus mous de voisinage et plus rarement une infiltration osseuse. Nous rapportons une observation. Il s’agit de Monsieur N.J. âgé de 67 ans, suivi pour une ostéomyélite chronique (OMC) du fémur droit fistulisée à la peau et évoluant depuis l’âge de 16 ans. Il s’est présenté actuellement pour des fistules productives, avec présence à l’imagerie d’une fracture pathologique sur une lésion ostéolytique de l’extrémité inférieure du fémur droit associé à une collection liquidienne mal limitée intra-médullaire étendue sur 8cm de grand axe. Une excision chirurgicale des fistules avec mise à plat de la collection et curetage de la cavité osseuse ont été réalisées. L’examen anatomopathologique a mis en évidence une prolifération tumorale maligne à type de carcinome épidermoïde différencié et kératinisant, infiltrant le trajet fistuleux avec extension au niveau des parties molles et infiltration de l’extrémité inférieure du fémur droit. Le bilan d’extension étant négatif, une désarticulation de la hanche a été pratiquée. Au dernier recule de deux ans, le patient va bien et ne présente pas de récidive locale ni de métastase à distance. La prise en charge initiale des ostéomyélites chroniques est primordiale pour prévenir les complications redoutables. En cas de transformation maligne et particulièrement en carcinome épidermoïde comme le cas de notre observation, l’amputation reste le traitement de choix pour prévenir les localisations secondaires.

## Abstract (en)

Malignant transformation of chronic osteomyelitis is a rare and late complication which mainly occurs at the level of the edges of a fistulous tract with extension and infiltration to the surrounding soft tissues and more rarely to bones. We here report the case of Mr N.J. aged 67 years, followed up for chronic osteomyelitis of the right femur fistulized to the skin and evolving since the age of 16 years. He presented with secerning fistulas. Imaging examination showed pathologic fracture due to osteolytic lesion of the lower extremity of the right femur associated with poorly defined intra-medullary collection of fluid measuring 8 cm along its longer axis. The patient underwent surgical resection of the fistulas with complete evacuation and curettage of the bone cavity. Pathological examination showed differentiated keratinising squamous cell carcinoma, infiltrating the fistulous tract and extending to the soft parts and to the lower extremity of the right femur. Staging was negative. Disarticulation of the HIP was performed. At two-years follow-up the patient was doing well without local recurrence or distant metastasis. Initial treatment of chronic osteomyelitis is essential to prevent alarming complications. Amputation is the treatment of choice in patients with malignant transformation of chronic osteomyelitis in particular to squamous cell carcinoma, as in the case of our patient, in order to to prevent secondary involvement.

## Introduction

La transformation maligne d’une ostéomyélite chronique est une complication rare et tardive [1]. Son mécanisme reste méconnu, mais on incrimine de plus en plus l’infection chronique, qui serait, selon certains auteurs, à l’origine de plus de 25% des processus néoplasiques [1]. Le passage à la chronicité est facilité par les caractéristiques histologiques de l’os ainsi que la capacité des bactéries à s’adapter à ce microenvironnement [2]. Cette transformation maligne survient le plus souvent au niveau des berges du trajet fistuleux.

## Patient et observation

**Figure 1:**
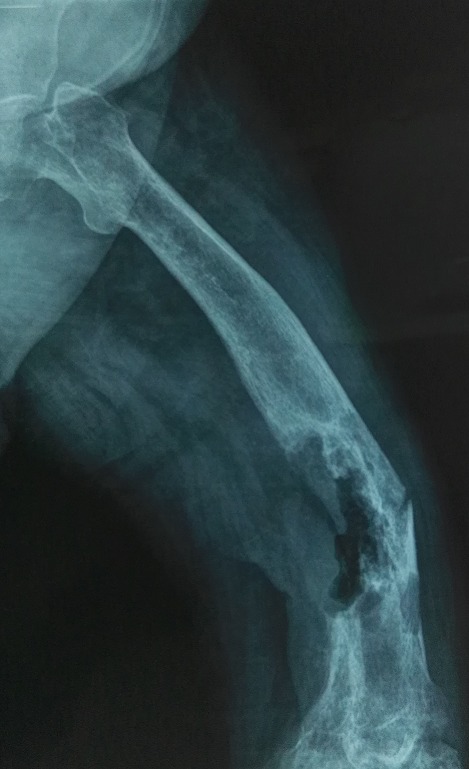
Radio standard: fracture pathologique au sein d’une lésion ostéolytique de l’extrémité inférieur du fémur droit

Il s’agit de Mr N.J âgé de 67 ans aux antécédents d’hypertension artérielle et dyslipidémie. Il a été hospitalisé à l’âge de 10 ans pour une ostéomyélite aigue. L’évolution a été marquée par le passage à la chronicité, après six années d’évolution, avec fistulisation à la peau. En 2015, le patient s’est présenté pour une douleur de la cuisse droite avec impotence fonctionnel. A l’examen, le patient présentait une tuméfaction inflammatoire de la cuisse droite avec fistule productive. A l’imagerie, la radiographie standard a montré une fracture pathologique au sein d’une lésion ostéolytique et un remaniement du fémur droit (Figure 1). A l’imagerie par résonance magnétique (IRM) on a objectivé une collection liquidienne mal limitée intra-médullaire et une extension à la loge musculaire postérieure et latérale externe sur 8 cm de grand axe. Il existait un contact étroit avec la veine fémorale superficielle. Le patient a initialement subi une excision du trajet fistuleux avec mise à plat de la collection postéro-externe et curetage de la cavité osseuse postérieure suivie d’une antibiothérapie et une immobilisation par atèle.

**Figure 2:**
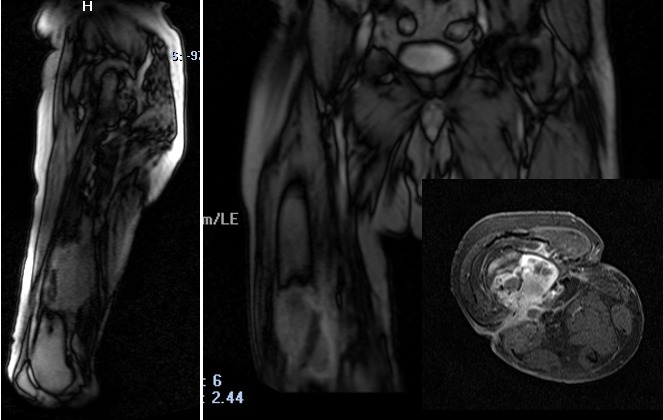
IRM: collection abcédée de la moitié inférieure de la diaphyse du fémur droit rompant la corticale postérieure sur une hauteur de 47 mm avec extension au niveau des parties molles postérieures

Un prélèvement des berges des trajets fistuleux et des prélèvements osseux et des parties molles ont été pratiqué. L’examen anatomopathologique a mis en évidence une prolifération tumorale maligne faite de massifs et amas cellulaires avec parfois des images d’enroulement et de kératinisation. Les cellules tumorales présentent des atypies nucléaires modérées et des mitoses nombreuses. Il existait quelques cellules dyskératosiques et certaines cellules tumorales à cytoplasme clarifié avec parfois des boules éosinophiles intra et extra-cytoplasmique. Le stroma est fibro-scléreux et peu inflammatoire. Cette prolifération tumorale est par place en continuité avec l’épiderme de surface qui est par ailleurs largement ulcérée, elle est accompagnée sur certains prélèvements des remaniements fibro-inflammatoires. Le diagnostic de carcinome épidermoïde différencié kératinisant infiltrant les parties molles et l’extrémité inférieure du fémur droit a été retenu. L’imagerie par résonance magnétique (IRM) de la cuisse droite (Figure 2) a mis en évidence une collection abcédée de la moitié inférieure de la diaphyse du fémur droit rompant la corticale postérieure sur une hauteur de 47mm, s’étendant au niveau des parties molles postérieures et refoulant les muscles ischio-jambiers, l’artère et la veine poplitées sus articulaires qui restent perméables. Cette collection est fistulisée à la peau avec deux trajets fistuleux actifs traversant les fascias inter-graisseux, l’un médial et l’autre latéral. Le pôle inférieur de cette lésion est situé à 6,6 cm de l’interligne articulaire fémoro-tibial, son pôle supérieur est situé à 18 cm de l’interligne fémoro-tibial. Le scanner thoraco-abdomino-pelvien ainsi qu’un scanner cérébral et une scintigraphie osseuse n’ont pas mis en évidence de localisation secondaire métastatique.

**Figure 3:**
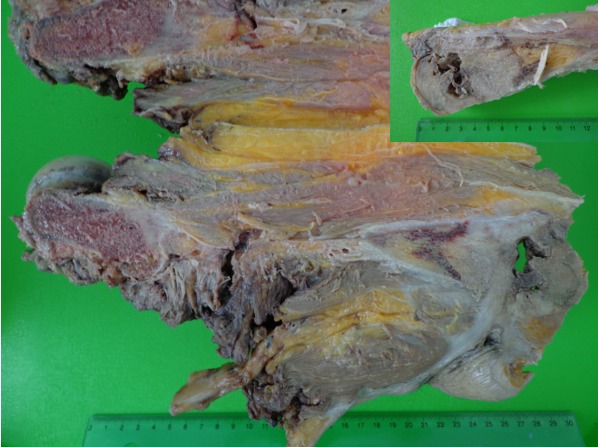
Macroscopie: induration cutanée en continuité en surface avec un orifice fistuleux et en profondeur avec une lésion tumorale qui infiltre l’extrémité inférieure du femur

**Figure 4:**
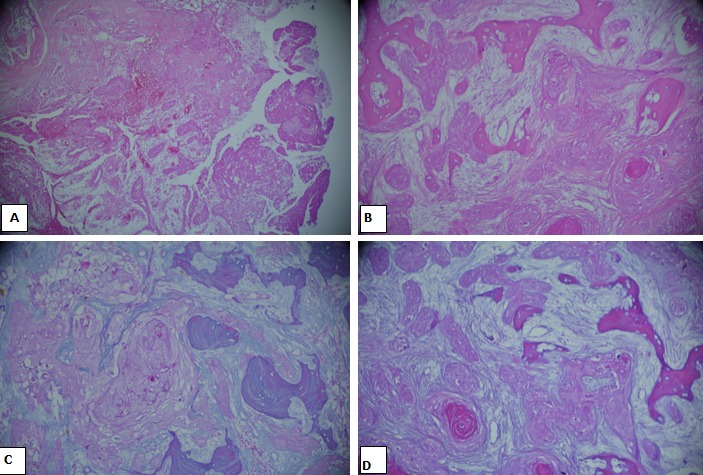
Histologie: prolifération tumorale maligne faite de massifs et amas cellulaires avec parfois des images d’enroulement et de kératinisation: A) aspect en regard du trajet fistuleux (HE x 100); B) infiltration osseuse (HE x 200); C) massifs et amas cellulaire avec des atypies nucléaires modérées et quelques cellules dyskératosiques (HEA50 x 400); D) images d’enroulement et de kératinisation (HEA50)

Le patient a eu une désarticulation de la hanche droite. L’étude macroscopique de la pièce opératoire a montré une induration cutanée qui siège à 8 cm de la limite chirurgicale proximale. Cette induration est en continuité en surface avec un orifice fistuleux et en profondeur avec une lésion tumorale qui détruit et infiltre l’extrémité inférieure du fémur et qui s’étend sur 6.5 x 6 cm. Cette lésion tumorale siège à 18 cm du col de fémur. Par ailleurs, le plan musculaire et sous cutané est sans autre lésion et en particulier sans autre lésion tumorale (Figure 3). L’étude histologique de la tumeur repérée à la macroscopie présente un aspect voisin de celui décrit sur la biopsie. Cette prolifération tumorale est par endroit en continuité avec l’épiderme, elle est ostéolytique et s’accompagne par place des foyers d’ostéogénèse réactionnelle (Figure 4). Il n’a pas été observé d’invasion vasculaire ni d’engainement péri-nerveux. Les limites chirurgicales sont saines. L’évolution en post opératoire précoce a été marquée par le développement d’une nécrose des berges qui ont nécessité une excision chirurgicale. L’évolution a été favorable sous antibiothérapie avec pansement. Au dernier recule de deux ans, le patient va bien et ne présente pas de récidive locale ou à distance.

## Discussion

La prévalence de la transformation maligne dans le cadre d’une OMC varie de 1.6 à 23% [1, 2] selon les séries. La moyenne d’âge au moment du diagnostic varie entre 50 et 60 ans [2], avec une nette prédominance masculine [1, 2]. L’intervalle libre entre le début d’OMC et la transformation maligne, varie entre 18 et 72 ans [1, 3, 4], il est de 51 ans dans notre cas. Les traumatismes et surtout les fractures ouvertes des os longs représentent la principale cause de l’OMC [1]. L’ancienneté de l’OMC serait donc le principal facteur impliqué dans le début de la carcinogenèse. Le type histologique le plus fréquent est le carcinome épidermoïde [1-3, 5]. Cette lésion touche le plus souvent le tibia et le fémur [1-3] et prend naissance à partir de l’épiderme au niveau des berges du trajet fistuleux puis infiltre les tissus adjacents y compris l’os [1].

L’augmentation du drainage des fistules, la persistance d’un ulcère chronique qui ne répond pas au traitement ou l’augmentation de sa taille, l’augmentation de la douleur et l’érosion ou la destruction progressive de l’os [2] peuvent être des signes alarmants suggérant une transformation maligne. Ces signes doivent inciter à la réalisation des prélèvements biopsiques et à l’étude histopathologique [6]. Un bilan d’extension complet clinique et radiologique doit être pratiqué en préopératoire et se base idéalement sur la tomographie avec émission de positrons (PET Scan) [7], il est dans la majorité des cas négatif, comme c’est le cas de notre observation. Le stade d’extension de la tumeur représente le principal facteur pronostic [1] et oriente l’attitude thérapeutique. L’amputation, pratiquée pour notre patient, reste le traitement de choix pour éviter la récidive locale et les localisations secondaires [3]. Ce traitement peut générer des troubles psychologiques et en particulier des troubles de l´image du corps ainsi que la dépression et des répercussions sur le plan social [2].

## Conclusion

La transformation maligne d’une OMC est rare mais doit être suspecté en présence des signes d’évolutivité chronique. L’étude anatomo-pathologique des prélèvements biopsiques est nécessaire au moindre doute. La prise en charge chirurgicale rapide après un bilan d’extension est obligatoire pour éviter la récidive locale et les localisations secondaires métastatiques. Cette complication redoutable rend compte de l’importance de la surveillance adéquate des OMC et la nécessité d’une biopsie au moindre doute clinique.

## Conflits d’intérêts

Les auteurs ne déclarent aucun conflit d'intérêts.
